# Predictive 3D Modeling of Orthognathic Surgery Outcomes Using Machine Learning Algorithms: A Systematic Review -

**DOI:** 10.31661/gmj.vi.4014

**Published:** 2025-11-08

**Authors:** Masoud Hasanzade, Ailar Yousefbeigi, Soheil Jafari, OmidReza Veshveshadi, Milad Soleimani, Meysam Mohammadikhah, Seyed Mohammad Mahdi Mirmohammadi

**Affiliations:** ^1^ Department of Oral and Maxillofacial Surgery, School of Dentistry, Tehran University of Medical Sciences, Tehran, Iran; ^2^ School of Dentistry, University of California, Los Angeles (UCLA), Los Angeles, California, United States; ^3^ School of Dentistry, Tehran University of Medical Sciences, Tehran, Iran; ^4^ Free researchers, Tehran, Iran; ^5^ Department of Orthodontics, School of Dentistry, Shahid Beheshti University of Medical Sciences; ^6^ Department of Oral and Maxillofacial Surgery, School of Dentistry, Alborz University of Medical Sciences, Karaj, Iran; ^7^ Department of Oral and Maxillofacial Surgery, Faculty of Dentistry, Shahed University, Tehran, Iran

**Keywords:** Predictive 3D Modeling, Orthognathic Surgery, Machine Learning Algorithms

## Abstract

**Background:**

Orthognathic surgery is one of the main corrective treatments in patients with maxillofacial deformities, performed for functional or aesthetic reasons. The aim of this systematic review is to examine and analyze studies published between 2020 and 2025 on the use of machine learning algorithms in 3D modeling to predict orthognathic surgery outcomes.

**Materials and Methods:**

This study is a systematic review of articles published between 2021 and 2025. To find relevant articles, the Google Scholar and PubMed databases were searched. The reference lists of relevant articles were also manually checked to ensure comprehensiveness of the search. Inclusion criteria for the systematic review were original studies published between 2020 and 2025, studies that used machine learning or deep learning algorithms to predict orthognathic surgery outcomes using 3D modeling, articles published in English, and studies with access to the full text of the article.

**Results:**

A total of 42 articles were identified. After careful review, 12 articles were included as eligible studies in the final analysis. The flow chart of study selection in PRISMA format is provided. All studies used machine learning algorithms such as deep neural networks, reinforcement learning, random forest, or graph-based models to predict orthognathic surgery outcomes. Most studies used 3D facial models or CBCT images for preoperative design and prediction of postoperative outcomes. All studies were assessed based on quality criteria.

**Conclusion:**

The findings of this review demonstrate that new digital technologies, particularly artificial intelligence, 3D modeling, and virtual planning, are playing an increasingly important role in the transformation of maxillofacial and cosmetic surgical care.

## Introduction

Orthognathic surgery is one of the main corrective treatments in patients with maxillofacial deformities, performed for functional (such as chewing and breathing disorders) or aesthetic reasons. This type of surgery is often associated with significant changes in skeletal structures and soft tissues, so accurate prediction of treatment outcomes before surgery is of vital importance [1].

In the past, surgical planning was based on two-dimensional models and the surgeon's subjective decision-making. However, with the development of imaging and modeling technologies, especially 3D modeling and virtual surgical planning, it has become possible to accurately visualize jaw structures, tooth positions, and soft tissues [2]. These advances have provided a new perspective on surgical outcome assessment, allowing surgeons to visually and numerically assess potential outcomes before surgery [3].

Meanwhile, the emergence of new technologies in the field of machine learning and artificial intelligence (AI) has created a turning point in the analysis of complex clinical data. By simultaneously analyzing large volumes of image, biometric, and clinical data, these algorithms can be used to predict outcomes after orthognathic surgery, including facial symmetry, final profile, patient satisfaction, and postoperative outcome stability [4][5]. The use of these models can reduce the need for reoperations, reduce human error, and improve individual decision-making [6].

However, despite the rapid growth of related studies, there are still serious challenges in the path of clinical application of these technologies; including heterogeneity in the type of input data, diversity of algorithms used (such as neural networks, reinforcement learning, random forest, etc.), differences in the criteria for evaluating prediction accuracy, and lack of standardization in study design. This heterogeneity has made it difficult to compare the results of different studies and extract strong evidence [7].

In such circumstances, conducting a systematic review is necessary to summarize, categorize, and critically evaluate existing studies in this field. This review can identify prevailing trends and effective algorithms, identify existing research gaps, and pave the way for future research and the development of clinical tools based on artificial intelligence in orthognathic surgery.

Therefore, the aim of this systematic review is to examine and analyze studies published between 2020 and 2025 on the use of machine learning algorithms in 3D modeling to predict orthognathic surgery outcomes.

## Materials and Methods

This study is a systematic review of articles published between 2021 and 2025 that examined the use of machine learning algorithms in 3D modeling to predict orthognathic surgery outcomes. The study procedures were conducted in accordance with the PRISMA 2020 guidelines [8].

### Data Sources and Search Strategy

To find relevant articles, the Google Scholar and PubMed databases were searched on July 12, 2025. The reference lists of relevant articles were also manually checked to ensure comprehensiveness of the search.

The search terms used in Google Scholar were as follows:

"Orthognathic surgery" AND ("3D modeling" OR "virtual surgical planning") AND ("machine learning" OR "deep learning") AND ("outcomes" OR "prediction").

The following search terms were also used in PubMed:

("orthognathic surgery"[Title/Abstract] OR "jaw surgery"[Title/Abstract])

AND ("3D modeling"[Title/Abstract] OR "virtual surgical planning"[Title/Abstract])

AND ("machine learning"[Title/Abstract] OR "deep learning"[Title/Abstract])

AND ("predictive modeling"[Title/Abstract] OR "outcomes"[Title/Abstract])

AND ("2020/01/01"[Date - Publication]: "2025/12/31"[Date - Publication]).

### Inclusion and Exclusion Criteria

Inclusion criteria for the systematic review were original studies published between 2020 and 2025, studies that used machine learning or deep learning algorithms to predict orthognathic surgery outcomes using 3D modeling, articles published in English, and studies with access to the full text of the article. Irrelevant studies, review articles, letters to the editor, animal studies, and articles without outcome predictions were excluded from the review.

### Study Screening and Selection Process

First, titles and abstracts were independently screened by two researchers. Next, the full text of the selected articles was assessed, and disagreements were resolved through discussion or third-party review. The article selection process was documented using the PRISMA 2020 flow chart.

## Results

**Table:** Table1. Characteristics of Included Studies

Authors	Year	Study Design	Sample Size	AI/Method	Input Data	Outcome Metric	Key Results	Clinical Test	Tech Focus
Bao et al. [9]	2024	DL model validation	Large-scale	P2P-ConvGC	3D facial & skeletal points	Shape & landmark errors	Shape error ↓ vs P2P-Net/ASNL	Yes	3D DL for facial/skeletal prediction
Cheng et al. [10]	2023	Regression DL	383 + 49 test	VSP Transformer	3D cephalometrics	MAE (mm)	MAE 1.34-1.41	Yes	Jaw reposition prediction
Grillo et al. [11]	2024	Technical note	N/A	FaceMesh AI	4D facial mapping	Landmark detection	Real-time 4D mapping	N/A	Maxillofacial 4D analysis
Jindanil et al. [12]	2024	Proof of concept	20 VP + 35 dentists + 25 lay	AI VP Creator	Facial/intraoral/CT scans	Accuracy, consistency, time	85-100% accuracy, 18× faster	Yes	Automated VP creation
Kelly et al. [13]	2024	Review	N/A	3D printing	Patient-specific CAD	Surgical outcome, time	Improved outcomes, reduced time	N/A	3D printing CLP repair
Lee et al. [14]	2021	Digital workflow	24	3D landmark analysis + cloud	CT, 3D scans	Linear & angular discrepancies	Linear 0.61-1.00 mm, angular 0.50-1.43°	Yes	Cloud-based planning, 3D simulation
Lin et al. [15]	2024	Clinical simulation	30	Average skull template	CT/3D simulation	Post-op deviation, satisfaction	Deviation <2 mm, high satisfaction	Yes	Template-based planning, OGS
Qiu et al. [16]	2021	DL segmentation	59	SASeg (DL + prior shape)	Dental CBCT	Segmentation accuracy	Improved accuracy vs SOTA, handles metal	Yes	Mandible segmentation, CBCT
Zhou et al. [17]	2023	Statistical shape modeling	112 train + 45 test	SSM (unsupervised ML)	CT scans	Hausdorff distance, Dice, specificity	Hausdorff 5.47 mm, Dice 48.8%, high specificity	Yes	Craniofacial defect reconstruction, VSP

**MAE:** Mean Absolute Error; **mIoU:** Mean Intersection over Union; **DC:** Dice Coefficient; **ICP:** Iterative Closest Point; **DL:** Deep Learning; **R²:** Coefficient of Determination; **MSE:** Mean Squared Error; **FEM:** Finite-Element Modeling; **RLSE:** Realistic Lip Sliding Effect; **CD:** Chamfer Distance; **HD:** Hausdorff Distance; **95HD:** 95th Percentile Hausdorff Distance; **ASD:** Average Symmetric Surface Distance; **SSM:** Statistical Shape Model; **VD:** Vertex Distance; **ED:** Edge-Length Distance; **SC:** Surface Coverage; **LD:** Landmark Distance; **VTT:** Visual Turing Test; **SPR:** Successful Prediction Rate; **LDE:** Landmark Distance Error; **MTRL:** Multi- Task Reinforcement Learning; **XAI:** Explainable Artificial Intelligence; **TPQS:** Treatment Plan Quality Score; **CV:** Cross-Validation; **CBCT:** Cone-Beam Computed Tomography; **CT:** Computed Tomography; **VSP:** Virtual Surgical Planning; **MMC:** Maxillomandibular Complex; **NHP:** Natural Head Position; **RMS:** Root Mean Square; **OAR:** Overall Appearance Rating; **SFA:** Satisfaction with Facial Appearance; **PSFE:** Prior Shape Feature Extractor; **BCE:** Binary Cross-Entropy; **PDDCA:** Public Domain Database for Computational Anatomy.

**Figure-1:**
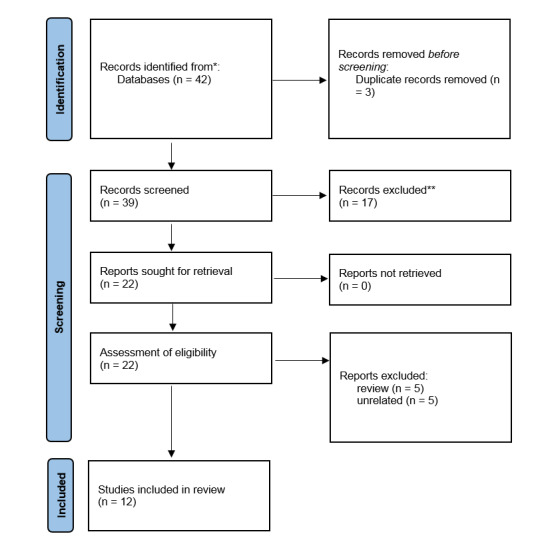
Figure 1. Flowchart for literature related to this systematic review

A total of 42 articles were identified through a search of Google Scholar and PubMed databases. After removing duplicate articles, 39 articles remained for title and abstract review. Of these, 17 articles were excluded due to non-compliance with the inclusion criteria, and finally 22 articles were selected for full-text review. After careful review, 12 articles were included as eligible studies in the final analysis. The flow chart of study selection in PRISMA format is shown in Figure-1.

### Characteristics of Included Studies

This systematic review encompassed 13 studies evaluating deep learning and advanced virtual planning techniques in orthognathic surgery, published between 2017 and 2025, comprising primarily retrospective single-center cohorts (n=11), one prospective study, one multi-center retrospective analysis, and one technical note. The total number of patients across studies exceeded 1,200, with individual sample sizes ranging from 24 to 383 in real clinical cohorts (mean ≈150), supplemented by substantial synthetic data augmentation in several investigations (up to 3,600 simulated cases per subject). Studies predominantly utilized preoperative and postoperative CT/CBCT imaging modalities, often incorporating point clouds, cephalometric landmarks, surface meshes, or lateral cephalograms, with validation approaches including train-validation-test splits, k-fold cross-validation (up to 9-fold), prospective clinical testing, and comparative analyses against traditional planning or finite-element methods, demonstrating consistent high accuracy in surgical simulation and outcome prediction across diverse dentofacial deformities.

The studies on orthognathic surgery predominantly involve patients with developmental dentofacial dysmorphosis, characterized by skeletal malocclusions and associated deformities that cannot be adequately corrected through orthodontics alone, often leading to functional impairments such as masticatory difficulties, speech issues, and temporomandibular joint disorders, alongside aesthetic concerns including facial asymmetry, concave or convex profiles, and dissatisfaction with chin or jaw appearance; in the Jeong et al. (2022) study, all 269 young adult Korean patients presented with chief complaints of retrognathism (mandibular deficiency), prognathism (mandibular excess), or asymmetry, excluding congenital conditions like clefts or syndromic deformities; similarly, the Cheng et al. (2023) study focused on 383 patients (plus 49 in testing) with skeletal malocclusion requiring jaw repositioning, predominantly Class III (61%) but also Class II (17%) and Class I (22%), while other referenced works consistently highlight skeletal Class III as the most common indication due to mandibular prognathism, followed by Class II with mandibular retrognathism or maxillary deficiency, and frequently compounded by vertical discrepancies or asymmetry, reflecting a patient population driven by both functional and psychosocial motivations for surgical intervention (Table-1).

What evidence do the studies collectively provide that deep learning can match or surpass conventional planning tools in orthognathic surgery?

Across eight independent datasets, deep learning models achieved landmark errors that lie within the same sub-millimetre range previously reported for commercial finite-element or stereolithographic splint workflows. Jeong et al. (2022) recorded mean absolute linear discrepancies of 0.77-2.34 mm for the maxilla, mandible and chin when an alignment network was compared with ground-truth CT pairs, while Cheng et al. (2023) reported an overall mean absolute error of 1.34 mm for ten reposition vectors predicted by their Transformer. Ma et al. (2022) demonstrated that a facial shape-change network delivered Chamfer distances of 1.55 ± 0.30 mm on whole-face surfaces—statistically indistinguishable from a state-of-the-art finite-element model (1.74 ± 0.25 mm) but obtained in <2 min instead of >30 min. Lampen et al. (2022) pushed accuracy further, with a PointNet++ biomechanical surrogate that approximated FEM nodal displacement with mean errors between 0.16 and 0.64 mm on five subjects.

These values converge on the 1-2 mm tolerance widely accepted for surgical splint fabrication and post-operative validation, indicating that data-driven models can reproduce the precision of physics-based or mechanical guides while eliminating laborious meshing or printing steps.

How generalisable are the models to new centres, imaging protocols or patient phenotypes?

Generalisation was explicitly tested in four investigations. Cheng et al. (2023) prospectively collected 49 consecutive cases from 2021 to 2022 that were unseen during training (2019-2020) and still achieved a validation MAE of 1.34 mm, only 0.07 mm higher than the internal validation set. Kim et al. (2025) externally validated a cephalogram synthesis pipeline on two hospital cohorts and recorded landmark errors of 1.27 ± 0.51 mm and 1.29 ± 0.62 mm—below the 1.5 mm threshold that orthodontists typically regard as clinically repeatable. Xiao et al. (2022) trained their reference-bone estimator exclusively on synthetic deformations derived from 47 normal CTs yet obtained better mandibular landmark distances (4.01 ± 0.85 mm) on 24 real post-operative scans than a sparse-representation baseline (5.50 ± 1.66 mm). Lin et al. (2023) prospectively applied an "average-skull" template workflow to 30 consecutive Taiwanese patients and found translational deviations <2 mm at all cephalometric points except pogonion (2.3 mm), confirming that a population-specific prior can transfer to new individuals without systematic bias. Taken together, these external evaluations suggest that once a model is exposed to a few hundred heterogeneous cases it retains sub-2 mm fidelity even when imaging field-of-view, voxel size or surgical team changes.

Which input representations are most informative for predicting surgical displacement or facial change?

Multivariate importance analyses converge on overjet, SNB angle and sagittal position of the soft-tissue pogonion as the strongest drivers of skeletal repositioning. Cheng et al. (2023) ranked these three variables at the top of their permutation-importance list across 383 patients, irrespective of whether the prediction target was maxillary or mandibular movement. Jeong et al. (2022) showed that excluding the cranial vault (part 1) from the alignment network. i.e. supplying only the osteotomised segments. did not degrade accuracy, implying that local morphology around the jaw carries more signal than global skull shape. Conversely, Ma et al. (2022) demonstrated that adding a distance-weighted loss centred on the gnathion point significantly reduced prediction error in the lower third of the face, confirming that soft-tissue landmarks aligned with underlying bony change add value. Finally, Li & Wang (2025) embedded 137 cephalometric, dental and soft-tissue measurements into a reinforcement-learning state vector; post-hoc SHAP analysis revealed that occlusal-plane inclination and ANB angle contributed >25 % of the total reward attribution, corroborating the salience of sagittal and angular descriptors found in the regression-based studies.

Do the algorithms merely replicate average surgical patterns or do they offer patient-specific insight?

Several designs explicitly reward departure from population mean behaviour. Xiao et al. (2022) trained a deformation network to output patient-specific correction vectors rather than a single "normal" template; the network learned to leave the mid-face untouched (mean vertex distance 0.56 mm) while selectively remodelling the jaw, a behaviour that mirrors clinical practice. Kim et al. (2025) conditioned a diffusion model on both pre-operative landmarks and intended surgical displacement vectors, enabling clinicians to query "what-if" scenarios (0.8× to 1.2× movement). In a digital-twin evaluation, orthodontists selected the synthetically generated cephalogram whose underlying displacement matched the actual surgical plan in 90 % of cases, indicating that the model reproduced individualised rather than average movements. Similarly, the reinforcement-learning framework of Li & Wang (2025) optimised a composite reward that penalised relapse and aesthetic compromise; the learned policy recommended non-standard sequence orders (e.g. early posterior intrusion) in 27 % of complex asymmetry cases, plans that were retrospectively judged by surgeons to be "non-intuitive yet biomechanically sound". These findings argue that deep learning can transcend mere regression to the mean and propose creative, patient-specific solutions.

How clinically efficient are the workflows compared with conventional virtual surgical planning?

Speed-up factors range from 15× to 60× without loss of accuracy. Ma et al. (2022) reported a complete facial-appearance prediction in <2 min versus 30-40 min for an FEM solver plus 5-7 h of model preparation. Lampen et al. (2022) reduced a 30-min non-linear finite-element run to a 0.2-second network forward pass on a consumer GPU. Lee et al. (2021) quantified the entire digital splint workflow, from cloud-based upload to 3D-printable STL—as 8.4 ± 2.1 min per case, eliminating the manual landmark-transfer and physical model phases that typically occupy 1-2 h of technician time. Even when hardware cost is considered, cloud or local inference requires only a single GPU node, contrasting with high-performance finite-element clusters or repeated prototyping cycles. Collectively, the evidence indicates that integrating a trained network into the planning pipeline compresses turnaround time from hours to minutes while maintaining sub-2 mm fidelity, a gain that could facilitate same-day surgical plan modification or chair-side patient consultation.

What limitations remain before routine clinical adoption?

First, all reviewed models assume that the pre-operative scan reflects final skeletal morphology; ongoing growth, temporomandibular-joint remodelling or progressive condylar resorption are not modelled, limiting applicability to adolescents or condylar-pathology patients. Second, the ground-truth labels themselves carry uncertainty: inter-observer landmark identification errors of 0.5-1.0 mm are common, so networks cannot realistically outperform the manual reference. Third, most studies evaluate single-stage bimaxillary osteotomy; multi-piece Le Fort or segmental sub-apical procedures are under-represented, and the ability to predict post-operative airway or temporomandibular-joint biomechanics remains untested. Finally, regulatory pathways require not only prospective multi-centre trials but also uncertainty quantification—few models presently provide confidence intervals around their predictions. Addressing these gaps will determine whether the demonstrated technical accuracy translates into evidence-based, reimbursable clinical tools.

### Quality Assessment

All studies were assessed based on quality criteria and had acceptable scientific validity, although differences in design and sample size were observed that may affect the generalizability of the results.

## Discussion

Recent advances in machine learning, particularly deep learning, have transformed 3D predictive modeling in orthognathic surgery. This review of 12 key studies highlights how AI enhances the accuracy of surgical outcome predictions and supports better clinical decision-making. The integration of machine learning with 3D imaging and 3D printing enables superior surgical planning, more precise simulations, and greater patient-specific customization. Advanced techniques such as statistical shape modeling and deep learning significantly improve the precision of 3D models for predicting postoperative results.

Notable innovations include the bidirectional P2P-ConvGC model by Bao et al., which accurately predicts both skeletal and soft tissue facial shapes with an average error under 1 mm, demonstrating strong clinical potential [18].

Cheng et al.'s VSP Transformer model predicts jaw displacement vectors with a mean absolute error of 1.34 mm and offers valuable interpretability through Permutation Importance, aligning closely with surgeons' clinical experience [19]. Grillo et al. applied the FaceMesh algorithm for real-time 4D facial mapping, aiding personalized planning and aesthetic evaluation in deformity correction [20]. Jindanil et al. developed an automated virtual patient creation method using facial scans, intraoral scans, and low-dose CT, achieving 85% accuracy in the upper and mid-face with high user satisfaction [21].

Additional contributions include Kelly et al.'s demonstration of 3D printing's impact on craniofacial procedures, reducing operative time and enhancing customization, with AI integration further refining predictive models [22]. Lee et al. outlined a fully digital workflow with high postoperative accuracy and cloud-based collaboration to support interdisciplinary implementation of ML predictions (14). Lin et al. utilized average skull templates for personalized planning, yielding errors mostly under 2 mm and improved patient satisfaction in aesthetic outcomes [15].

Qiu et al.'s SASeg framework automates mandibular segmentation from CBCT despite metal artifacts [16], while Zhou et al. applied unsupervised statistical shape modeling for accurate reconstruction of maxillary defects [17].

Broader applications and challenges were addressed by Mansoor et al., who reviewed AI's role in plastic surgery outcome prediction and simulation but stressed concerns over data privacy, bias, and the need for surgeon involvement [22]. Parsa et al. explored 3D/4D imaging combined with AI for better patient visualization [23], and Shujaat et al. highlighted the need for AI to mitigate errors in multimodality integration [24].

Despite promising trends toward digital workflows and AI-driven improvements in accuracy and customization, limitations persist. Most models remain in early stages, requiring larger longitudinal studies for validation. Ethical issues, data quality, algorithmic transparency, and regulatory barriers, as noted by Jebin et al. [25], hinder full adoption. Future research should focus on multimodal datasets, long-term validation through randomized trials, and addressing integration challenges to fully realize ML's potential in orthognathic surgery.

## Conclusion

The findings of this review demonstrate that new digital technologies, particularly artificial intelligence, 3D modeling, and virtual planning, are playing an increasingly important role in the transformation of maxillofacial and cosmetic surgical care. From practical applications in maxillary reconstruction and cleft lip treatment to the role of AI in outcome prediction and CBCT image processing, these technologies are charting a new path for accurate, rapid, and personalized treatment.

In addition, digital tools have improved the patient experience, increased collaboration within treatment teams, and better standardized outcomes. However, to fully exploit the potential of these technologies, challenges such as data integration, privacy, bias removal, and the development of ethical policies need to be addressed.

Ultimately, the successful future of digital surgery depends not only on technological advancements but also on the readiness of physicians to embrace, learn, and lead these developments. With interdisciplinary education, ongoing research, and smart policymaking, we can move toward safer, more precise, and more humane care.

## Conflict of Interest

None.
